# Salinity tolerance in wheat: rethinking the targets

**DOI:** 10.1093/jxb/eraf152

**Published:** 2025-04-09

**Authors:** Sergey Shabala, Xi Chen, Ping Yun, Meixue Zhou

**Affiliations:** School of Biological Science, University of Western Australia, Crawley, WA6009, Australia; International Research Centre for Environmental Membrane Biology, Foshan University, Foshan 528000, China; Crop Physiology, School of Life Sciences, Technical University of Munich, Freising 85354, Germany; School of Biological Science, University of Western Australia, Crawley, WA6009, Australia; Tasmanian Institute of Agriculture, University of Tasmania, Launceston, TAS 7250, Australia; Sher-e-Kashmir University of Agricultural Sciences and Technology of Kashmir (SKUAST-Kashmir), India

**Keywords:** Autophagy, H^+^-ATPase activity, potassium cycling, purine metabolism, stress signaling, vacuole, xylem loading

## Abstract

Wheat is a major staple food in the human diet, but its production under current climate scenarios is problematic given the predicted extent of land salinization and the fact that wheat is highly sensitive to soil salinity. This work aims to critically assess previous breeding efforts and the pros and cons of targeting Salt Overly Sensitive 1 (*SOS1*) and *High-affinity K*^*+*^  *Transporter 1* (*HKT1*) genes to improve salinity stress tolerance in wheat. We argue that overexpressing *SOS1* genes encoding Na^+^/H^+^ exchangers for Na^+^ removal from root to the rhizosphere may come with the caveat of increased loading of Na^+^ into the xylem and its delivery to the shoot, as well as numerous pleiotropic effects. Similarly, targeting HKT1 transporters for removing Na^+^ from the shoot comes with significant yield penalties due to the high carbon cost of osmotic adjustment; this strategy is also limited by the relatively small capacity of the root to store excessive Na^+^ without experiencing toxicity symptoms. We suggest that targeting tissue tolerance traits such as K^+^ retention in mesophyll and vacuolar Na^+^ sequestration in the shoot will be able to deliver better outcomes. We also call for a better understanding of the structure–function relationships of various isoforms of key proteins involved in maintenance of Na^+^ and K^+^ homeostasis and a need for more in-depth physiological studies of wheat species with the DD genome, a key contributor to tissue tolerance traits. Our arguments are supported by a bioinformatic analysis of the number of orthologs for some key genes between hexaploid (AABBDD) and tetraploid (AABB) wheats and their structural differences.

## Introduction

Wheat (*Triticum* spp.) is the major staple food for over one-third of the world’s population and provides 20% of the calories and protein in the global human diet (The International Wheat Genome Sequencing Consortium, 2014; Shewry and Hey, 2015). Two major types of domesticate wheat exist: durum wheat (*Triticum durum*; tetraploid; AABB genomes) and bread wheat (*Triticum aestivum*; hexaploid; AABBDD genomes) (Supplementary Fig. S1). Wheat production needs to be doubled by 2050 to match population growth (Kotula *et al.*, 2024) but this task is rather challenging, given the growing extent of soil salinization (Liu *et al.*, 2020) and the fact that wheat is classified as a salt-sensitive species (Munns and Tester, 2008). Indeed, a 10% drop in grain yield of durum wheat is observed in salinities as low as 4.7 dS m^−1^; for bread wheat this threshold is slightly higher (6.7 dS m^−1^; Wu *et al.*, 2015b and references therein). As a result, when grown under saline soil conditions typically found in agricultural production systems (equivalent to 100–150 mM NaCl), many wheat varieties fail to produce any grain, and even the most tolerant ones show yield penalties as high as 85% (Houshmand *et al.*, 2005; Zhu *et al.*, 2016b). Despite numerous efforts (a search on Web of Science using key words ‘wheat’ and ‘salinity’ reveals over 8800 entries), the progress in wheat breeding for salinity tolerance has been rather modest. This work aims to understand the reasons behind this lack of progress in the field. To do this, we summarize some of the most popular traits used in previous studies and discuss their pros and cons as targets in wheat breeding programs. Our focus is on comparing performance of two key domesticated wheat species—durum and bread wheat—and revealing physiological and genetic mechanisms conferring differential salt tolerance between them.

### Should we target SOS1 transporters for Na^+^ exclusion?

Experiments with radiolabeled ^22^Na^+^ probes have shown that differential tolerance to salt stress between durum and bread wheat is not related to unidirectional uptake of Na^+^ by roots (Davenport *et al.*, 1997). The same was true for genetic variability in salinity tolerance within the same species. In durum wheat, genotypes with contrasting salinity tolerance did not differ significantly in unidirectional root uptake of Na^+^ and there was no evidence for recirculation of Na^+^ from shoots to roots (Davenport *et al.*, 2005). These findings are fully consistent with data on other cereal crops, such as barley, where no significant difference was found between contrasting cultivars in their unidirectional ^22^Na^+^ influx (Chen *et al.*, 2007). In the light of this, differential salinity stress tolerance in crops (including wheat) is generally attributed to either the ability of a genotype or species to exclude Na^+^ back to the rhizosphere [by Na^+^/H^+^ exchangers encoded by *Salt Overly Sensitive 1* (*SOS1*) gene] or their ability to retrieve Na^+^ from the shoot [mediated by High-affinity K^+^ Transporter 1 (HKT1) transporters] (van Zelm *et al.*, 2020; Zhao *et al.*, 2020).

Munns (2005) estimated that about 98% of all Na^+^ taken up by wheat roots is returned to the rhizosphere via an active Na^+^ extrusion; this process is believed to be mediated by a Na^+^/H^+^ antiporter encoded by *SOS1* gene (Zhao *et al.*, 2020). Discovered first in Arabidopsis, the SOS pathway is now considered as the main (and only) mechanism for thermodynamically active Na^+^ extrusion. The SOS module consists of three elements: the SOS1 Na^+^/H^+^ antiporter *per se* that exchanges 1 Na^+^ for 1 H^+^, and two regulatory elements, SOS2 [CIPK24; a CBL interacting protein kinase 24 belonging to the sucrose non-fermenting 1 (SNF1)/AMP kinase (AMPK) family; Halfter *et al.*, 2000] and SOS3 (CBL4, a calcineurin B like protein 4). It was shown that CaMV 35S promoter-driven overexpression of the Arabidopsis *SOS1* gene improved plant salt tolerance (Shi *et al.*, 2003), while a mutant, *sos1*, had a salt-sensitive phenotype (Oh *et al.*, 2010). *SOS1* expression was reported to be significantly up-regulated by salt stress in roots of various plant species (e.g. radish—Zhang *et al.*, 2021; perennial ryegrass—Liu *et al.*, 2018; Brassica—Nutan *et al.*, 2018), and a positive correlation between SOS-related gene expression and differential salt tolerance between cultivars was reported for some species, including wheat (Sathee *et al.*, 2015).

Given the traditional view of Na^+^ exclusion being key for improving salinity tolerance, it is hardly surprising that numerous attempts have been made to overexpress genes related to the SOS pathway to improve plant phenotype. Positive examples include a beneficial role of *SOS1* overexpression for salinity tolerance in radish (Zhang *et al.*, 2021), *Lilium pumilum* (Yang *et al.*, 2023), cotton (Xu *et al.*, 2023), soybean (Nie *et al.*, 2015), and poplar (Zhou *et al.*, 2014). Attempts were also made to express *SOS1* genes from some halophyte species in crops to boost salt tolerance. For example, overexpression of *NsSOS1* from *Nitraria sibirica* improved salt tolerance of transgenic poplars (S. Chen *et al.*, 2024), while overexpression of *TrSOS1* from the recretohalophyte *Tamarix* in Arabidopsis wild type and *sos1* mutant could significantly improve transgenic plant growth and reduce Na^+^ content (Cheng *et al.*, 2019). Overexpression of the constitutively active form of *SOS2* (in poplar; Yang *et al.*, 2015) or *SOS3* (in Arabidopsis; Yang *et al.*, 2009) also increased salt tolerance.

To our great surprise, we were unable to find a single paper reporting success in overexpressing genes from the SOS pathway to improve salinity stress tolerance in wheat species. Given the number of salinity-related studies on wheat, it is difficult to believe that this may be merely an omission and that no such attempts were made. Most likely, the results of such overexpression were either negative or inconclusive, explaining the lack of publication. Is this true? And if so, why? Several key aspects will be discussed here.

#### Constitutive overexpression of *SOS1* may increase Na^+^ load in the shoot

In addition to being present in root epidermis (where it operates in Na^+^ exclusion to the rhizosphere), SOS1 is also very abundant in root stele (xylem parenchyma tissue) and, as such, may contribute to xylem Na^+^ loading. Reported first by β-glucuronidase staining in Arabidopsis (Shi *et al.*, 2002), this notion was then confirmed in numerous physiological experiments on other species. Yadav *et al.* (2012) showed that overexpression of *SbSOS1* gene from the extreme halophyte *Salicornia brachiata* enhances Na^+^ loading in xylem in transgenic tobacco. Similarly, overexpression of *SlSOS2* (*SlCIPK24*) in *Solanum lycopersicum* resulted in higher Na^+^ content in stems and leaves in transgenic tomato (Huertas *et al.*, 2012). Thus, a constitutive overexpression of *SOS1* may be considered a ‘double-edged sword’ and could negate all beneficial results of Na^+^ extrusion into the rhizosphere by a concurrent increase in the amount of Na^+^ loaded into the xylem and transported to the shoot. If the latter hypothesis is true, then more tolerant wheat species should either possess a lower number of SOS1 transporters at the xylem parenchyma or, at the very least, have epidermal and stelar SOS1 transporters that are regulated differently.

To provide some further insights into this issue, we have undertaken a bioinformatic approach using Orthofinder (Emms and Kelly, 2019) to identify the amount of SOS1 isoforms in tetraploid (durum wheat and wild emmer) and hexaploid (bread wheat and spelt; genetic lineage shown in Supplementary Fig. S1) wheats. While one would expect that a higher ploidy level would result in a larger number of isoforms, this was not the case for SOS1, with the average number of SOS1 orthologs in tetraploid wheats being 3-fold higher than that in hexaploid wheats (significant at *P*<0.05). Thus, higher levels of SOS1 orthologs correlate negatively with salt tolerance (as both bread wheat and spelt are considered much more tolerant than the two other species; Colmer *et al.*, 2006; Munns *et al.*, 2006).

#### SOS1 exchanger may have many other (potentially pleiotropic) roles

Earlier Oh *et al.* (2010) showed that in addition to higher accumulation of Na^+^, an *Atsos1-1* mutant showed inhibition of endocytosis, abnormalities in vacuolar shape and function, and changes in intracellular pH compared with the wild type in root tip cells under stress. Also affected were expression levels of root-specific Ca^2+^ transporters including several CAXs (cation exchangers) and cyclic nucleotide-gated channels (CNGCs). The latter are Na^+^ and K^+^ permeable and, thus, could strongly affect plant ionic homeostasis. Consistent with this, Shabala *et al.* (2005) used electrophysiological approach to demonstrate that SOS mutations also affect H^+^-ATPase activity and intracellular K^+^ homeostasis, with major implications for salt tolerance. Taken together, these results suggested that SOS1 protein, in addition to its function as a Na^+^/H^+^ antiporter, may have other roles, whose disruption affected membrane traffic, vacuolar functions, and potassium homeostasis. Also, in addition to affecting SOS1 operation, SOS2 and SOS3 interact with the plasma membrane transporter PUT3 involved in polyamine transport (Chai *et al.*, 2020). At the same time, polyamines regulate a variety of cation and K^+^-selective channels as well as activities of Ca^2+^- and H^+^-ATPases (Pottosin *et al.*, 2014a), with major implications for plant adaptive responses to salinity (Pottosin *et al.*, 2014b). Thus, targeting SOS1 in breeding programs may lead to numerous pleiotropic results not necessarily beneficial for plants.

#### Regulation of SOS1 is complex and likely to be tissue specific

**Fig 1.**
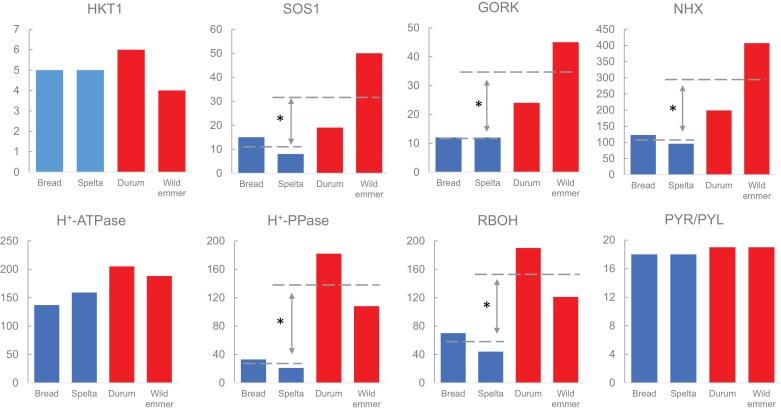
Difference in the number of orthologs for some key gene between hexaploid (AABBDD; in blue) and tetraploid (AABB; in red) wheats. The analysis was performed using OrthoFinder (Emms and Kelly, 2019), utilizing genome-wide protein sequences obtained from Ensembl Plants, which include all gene isoforms. Gene families including NHX, H^+^-ATPase, H^+^-PPase, and RBOH, were identified based on conserved domains using hidden Markov model (HMM) profiles from the InterPro database (NHX, PF00999; H^+^-ATPase, PF01496; H^+^-PPase, PF03030;RBOH, PF08414) and analysed with hmmscan (version 3.4) under default parameters (Finn *et al.*, 2011). Other gene candidates, including HKT1, SOS1, GORK, and PYR/PYL, were inferred from orthogroups identified by Orthofinder (X. Chen *et al.*, 2024).

The canonical view on SOS1 operation is that it is autoinhibited under normal conditions (Zhao *et al.*, 2020). SOS1 forms a dimer, with an NhaA-folded transmembrane domain portion in the membrane and an elongated cytosolic portion of multiple regulatory domains in the cytoplasm (Zhang *et al.*, 2023), and switches from an occluded conformation in the auto-inhibited state to a conducting conformation in the active state via a conformational transition of the highly conserved Pro148 in the unwound transmembrane helix 5 (TM5). This inhibition is released by the phosphorylation of Ser1044 at the C-terminal domain of SOS1 by SOS2 under salt stress (Quintero *et al.*, 2011). In its turn, SOS2 is recruited to the plasma membrane by SOS3 via its interaction with the regulatory domain of SOS2. Under normal conditions, the kinase activity of SOS2 is inhibited by 14-3-3 and GIGANTEA (GI) proteins (Duscha *et al.*, 2020; Zhao *et al.*, 2020). Salt stress promotes the degradation of 14-3-3 and GI, resulting in the release of SOS2 from SOS2–GI/14-3-3 complexes and consequently the activation of SOS2 by SOS3 (Kim *et al.*, 2013; Zhou *et al.*, 2014). Recently, Xie *et al.* (2024) showed that CBL5, an ortholog of CBL4 and CBL10 in Arabidopsis, also interacts with and recruits CIPK8/CIPK24 to the plasma membrane. Regulation of SOS1 operation may be different in roots and shoots, as shown by Yin *et al.* (2020). However, to the best of our knowledge, no comparison has been made on the difference in regulation of SOS1 operation between root epidermal and stelar tissues. Also, given the large number of SOS1 orthologs (Fig. 1), it would be reasonable to suggest that their regulation may differ, explaining tissue-specific operation of SOS1 exchangers.

**Fig 2.**
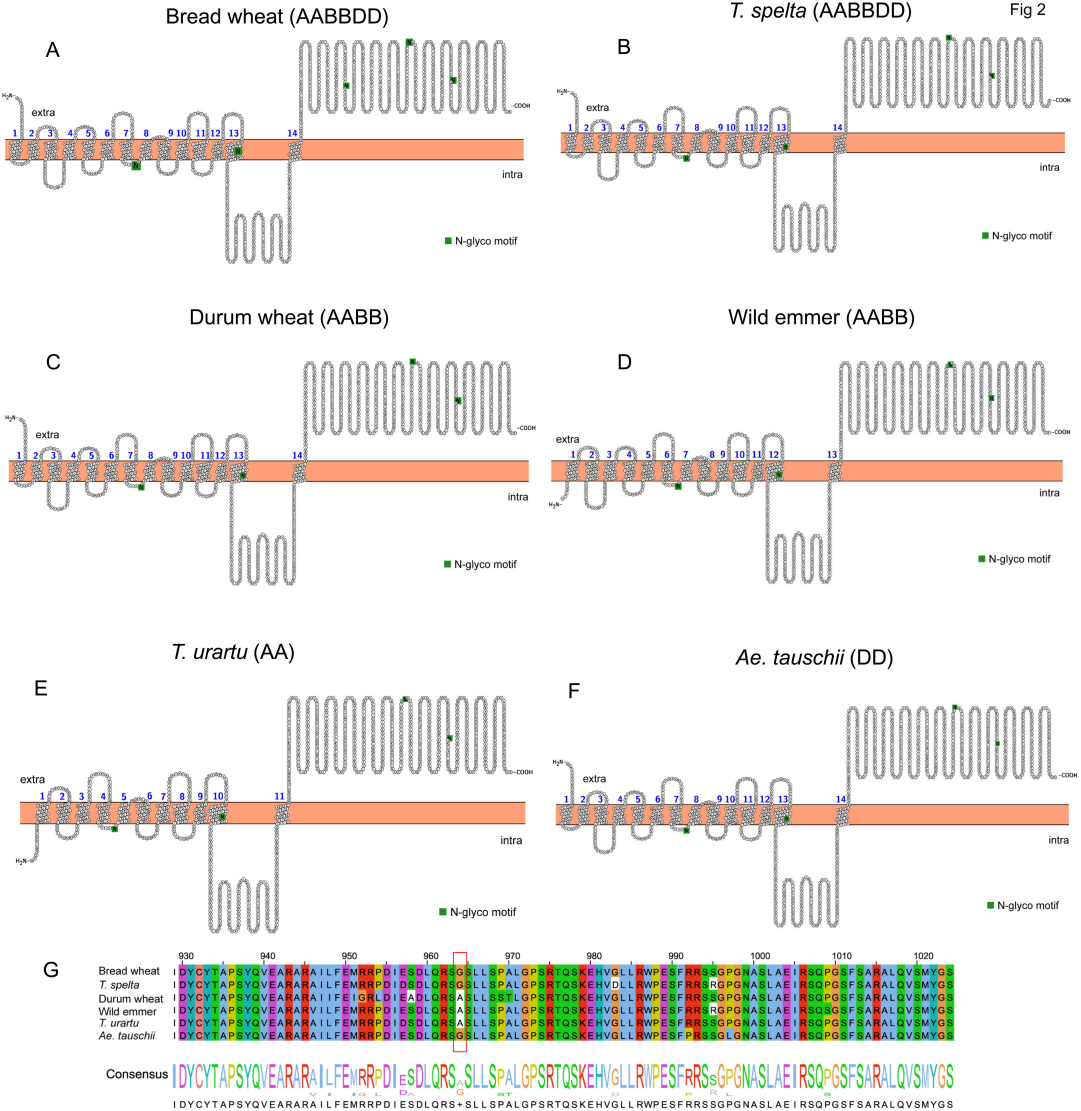
Differences in SOS1 structure amongst wheat relatives. (A–F) Predicted transmembrane topologies of SOS1 proteins in six species (*Triticum aestivum*, hexaploid, AABBDD; *Triticum spelta*, hexaploid, AABBDD; *Triticum durum*, tetraploid, AABB; *Triticum dicoccoides*, wild emmer, tetraploid, AABB; *Triticum urartu*, diploid, AA; *Aegilops tauschii*, diploid, DD). The SOS1 protein sequences from six species were identified based on OrthoFinder results and BLAST comparison with the TaSOS1 protein sequence obtained from Ensembl Plants. The transmembrane helices and *N*-glycosylation sites were predicted using Protter (Omasits *et al.*, 2013). (G) Sequence alignment of SOS1 (amino acid residues 930–1023) across six species (Katoh *et al.*, 2002). Sequences with the highest similarity to TaSOS1 were selected for alignment via BLASTP. An all-against-all BLASTP search was conducted, and results were filtered with an E-value<10^−10^.

To further provide insights into the possible differences in regulation of SOS1 transporters as a basis for differential salinity tolerance between tetra- and hexaploid wheats, we have compared the predicted topology and structure of SOS1 exchangers using Protter (Omasits *et al.*, 2013), predicting the transmembrane regions and *N*-glycosylation motifs, and aligned the SOS1 protein sequences using MAFFT (Fig. 2) (Katoh *et al.*, 2002). The general structure of SOS1 was similar between bread and durum wheat, except that bread wheat had one extra protein *N*-glycosylation motif. The latter plays an important role in proper protein folding and stability in the endoplasmic reticulum, as well as in trafficking the protein to the correct cellular compartment (Nagashima *et al.*, 2018). A moderate number of *N*-glycosylation sites can regulate the efficiency of ion transport by the protein (Li *et al.*, 2024), and it was shown that mutants defective in *N*-glycan maturation are more salt-sensitive than wild type because *N*-glycosylation is important for cell wall formation under salt stress (Kang *et al.*, 2008). Another interesting pattern was a cytosolic location of the N-terminal end of SOS1 in two progenies of durum wheat, a wild emmer, and *Triticum urartu* (Fig. 2) while in all species containing a DD genome (bread wheat, spelt, and *Aegilops tauschii*) it was located in the apoplastic space. The number of transmembrane domains was also much lower in the BB compared with the DD genome—11 in *T. Urartu*, 13 in wild emmer, compared with 14 in all three DD species (Fig. 2). Finally, differences were found in the structure within the motif of the large cytosolic domain (box in Fig. 2G), with a replacement of G by A in species with the DD genome. It was shown before that a difference in selectivity of two types of HKT transporters (and their preferential transport of Na^+^ in a type I HKT) is determined by a substitution of a single amino acid residue (S versus G) in a selectivity filter (Mäser *et al.*, 2002). It is tempting to speculate that a similar replacement of G^964^ by A^964^ may also confer a difference in regulation of SOS1 transporters between DD and non-DD genome wheats.

### Can HKT1 be a ‘silver bullet’ for improving salinity tolerance in wheat?

Traditionally, a higher sensitivity of durum wheat compared with bread wheat is explained by the lack the Na^+^-excluding locus *Kna1* found on the D genome and attributed to the operation of the HKT1 transporter (Byrt *et al.*, 2007; James *et al.*, 2011; Yang *et al.*, 2014). The latter belongs to the Transporter of K^+^ (TrK)/K^+^ transporter (Ktr)/HKT family that is implicated in various functions, from K^+^ or Na^+^ uptake to maintenance of membrane potential, ion homeostasis, and Na^+^ recirculation from shoot to root (Corratgé-Faillie *et al.*, 2010). The HKT family can be divided into two distinct sub-families based on their ion selectivity (Mäser *et al.*, 2002; Venkataraman *et al.*, 2021), and transporters in sub-family I, such as TmHKT1;5 in wheat, AtHKT1;1 in *Arabidopsis*, or OsHKT1;1 and OsHKT1;5 in rice, are highly selective for Na^+^ (Platten *et al.*, 2006) and operate in Na^+^ retrieval from the shoot. Given the notion that Na^+^ toxicity in the shoot is a key factor reducing crop performance under saline conditions (van Zelm *et al.*, 2020), it is hardly surprising that HKT1 transporters have long been an extremely attractive target for crop genetic improvement.

In durum wheat, the *TmHKT1;5-A* gene was shown to be present in the *Nax2* quantitative trait locus (QTL), which contributes to lowering Na^+^ levels in leaves (Byrt *et al.*, 2007; Munns *et al.*, 2012). Although *HKT1;5-A* is present in *Triticum monococcum*, it has not been found in any accession of *Triticum urartu*, which is the ancestral donor of genome A for modern durum and bread wheat (Munns *et al.*, 2012). The *Nax2* region on 5AL is homoeologous to the region on chromosome 4DL containing *Kna1*, the major Na^+^ exclusion locus in bread wheat (Byrt *et al.*, 2007). Another important allele for Na^+^ exclusion, *Nax1*, was also identified in wheat by QTL analysis (James *et al.*, 2006). In contrast to *Nax2*, this locus was able to confer Na^+^ unloading not only in roots but also in leaf sheaths. Later, Munns *et al.* (2012) overexpressed *TmHKT1;5-A* gene in a near-isogenic line (NIL) and compared its performance with its parental cultivar, Tamaroi, lacking functional *TmHKT1;5-A* within the *Nax2* locus. The authors reported a 25% increase in grain yield in associated with reduction in Na^+^ content in the flag leaf. Yang *et al.* (2014) compared the salt tolerance of a synthetic allohexaploid wheat (neo-6x) with its tetraploid (*Triticum turgidum*; BBAA) and diploid (*Aegilops tauschii*; DD) parents. They showed that expression of the D-subgenome *HKT1;5* homologue resulted in a shift from the constitutive high basal expression in *Ae. tauschii* (2*x*) to salt-induced expression in neo-6*x*. This was consistent with the findings of a genome-wide association study by Babgohari *et al.* (2013), who showed that the D-genome allele (*HKT1;5-D*) showed higher expression than the A genome allele (*HKT1;5-A*) when subjected to a high NaCl level.

Variation in copy number for individual HKT gene members was observed between the barley, wheat, and rice genomes (Huang *et al.*, 2008) prompting a question of whether the number of HKT1 orthologs may be a factor contributing to differential salinity stress tolerance. This seems not to be the case, as evident from our comparison of HKT1 orthologs in tetra- and hexaploid wheats (Fig. 1). Thus, protein structural changes due to amino acid changes may play a significant role in the latter process. It was shown that structural variations in HKT1;5 may underpin differences in Na^+^ transport capacity, with differences in residues at D-471/A and D-474/G(473) determining different affinities for Na^+^ between *T. monoccocum* (TmHKT1;5-A) and *T. aestivum* (TaHKT1;5-D) (Xu *et al.*, 2018). The same was also true for rice, where Somasundaram *et al.* (2020) showed that allelic variation in OsHKT1;5 sequence in specific landraces correlated with variation in salt tolerance, with four key amino acids in the loops on the extracellular side (E239K, G207R, G214R, L363V) conferring both affinity and Na^+^ conductance of the transporter.

While reducing cytosolic Na^+^ content in metabolically active photosynthetic tissues in the shoot is indeed critical for optimal plant performance, the strategy of excluding Na^+^ from uptake comes with the caveat of a compromised osmotic adjustment (Shabala, 2013). Indeed, the cost of production of compatible solutes (organic osmolytes) required for osmotic adjustment and turgor maintenance in salt-grown plants is at least an order of magnitude higher than the cost of sequestering toxic Na^+^ in cell vacuoles (Munns and Gilliham, 2015; Munns and Millar, 2023), drastically reducing availability of the ATP or carbon pool for processes necessary for maintenance (e.g. protein turnover, synthesis of lipids and carbohydrates, maintenance of ion gradients) and growth (e.g. nutrient acquisition, source to sink transfer). Thus, reliance on HKT1-mediated Na^+^ exclusion will inevitably come with yield penalties associated with the high cost of osmotic adjustment. The above work by Munns *et al.* (2012) is a good illustration of that fact: despite a 25% yield increase in NIL lines grown under saline conditions, these plants still showed a 50% yield reduction compared with (non-saline) controls. Reliance on Na^+^ as a cheap osmoticum is a strategy validated by millions of years of evolution in halophytes (Flowers *et al.*, 2015), and also employed by some of the most tolerant crops. For example, Huang *et al.* (2019) showed that HvHKT1;5 negatively regulates salt tolerance in barley, with a knockdown *Hvhkt1;5* line showing improved salt tolerance. Even in rice, t-DNA tagging-based gain-of-function of *OsHKT1;4* reinforced Na^+^ exclusion from leaves and stems but triggered Na^+^ toxicity in roots under salt stress, resulting in a sensitive phenotype. From the physiological point of view, this is hardly surprising. The Casparian strip in the root is impermeable to ions; thus, all Na^+^ removed from the shoot would be accumulated in the stele. As the latter has a very limited volume (a few orders of magnitude smaller that the shoot volume), toxic Na^+^ effects in the root following the operation of HKT1 transporters are inevitable.

Similar to SOS1, overexpression of HKT1 transporters may also come with pleiotropic effects, both positive and negative. Dissanayake *et al.* (2024) showed that differential expression of *HKT1;4* and *HKT1;5* genes in bread wheat varieties resulted in metabolic re-arrangements in energy conversion, primary metabolic machinery, phenylpropanoid pathway, and root lignification. It was also shown that *Nax* loci (hosting HKT1 transporters) confer two highly complementary mechanisms, both of which contribute towards reducing the xylem Na^+^ content. One enhances the retrieval of Na^+^ back into the root stele via HKT1;4 or HKT1;5, whilst the other reduces the rate of Na^+^ loading into the xylem via SOS1 (Zhu *et al.*, 2016a). Thus, the actual role of HKT1 in Na^+^ unloading from the xylem may be not as straightforward as previously believed (Alnayef *et al.*, 2020), and its targeting in breeding programs should be done with great caution.

### Potassium retention in mesophyll: a largely unexplored trait

Over the last 15 years, the essentiality of K^+^ retention has emerged as a key element conferring salinity tissue tolerance (Wu *et al.*, 2018). Discovered first for barley roots (Chen *et al.*, 2005, 2007), this trait has since been reported for many plant species including wheat (Cuin *et al.*, 2008, 2010). This trait also explains the inter-specific variability in salinity stress tolerance (e.g. poplar—Sun *et al.*, 2009; mangroves—Lu *et al.*, 2013; *Brassica*—Chakraborty *et al.*, 2016) as well as conferring differential salinity stress tolerance between halophytes and glycophytes (Himabindu *et al.*, 2016; Percey *et al.*, 2016; Mann *et al.*, 2021). More recently, cytosolic K^+^ retention in a shoot has also emerged as a novel (and essentially overlooked) mechanism that explain intra- and inter-specific variability in salinity stress tolerance (Wu *et al.*, 2014; Niu *et al.*, 2018). This is specifically true for wheat. By screening 48 wheat genotypes, Wu *et al.* (2014) showed a 2-fold difference in cytosolic K^+^ retention in mesophyll observed between bread and durum wheats, with lesser NaCl-induced K^+^ loss reported for bread wheat. An essential role of leaf K^+^ retention as a determinant of salinity stress tolerance was also reported for durum wheat by Soni *et al.* (2021). Thus, differential salt tolerance between these two species cannot be attributed merely to operation of HKT1 transporter and better Na^+^ retrieval from the shoot.

Electrophysiological and genetic studies demonstrated that depolarization-activating outward-rectifying K^+^ channels encoded by the *GORK* gene represent the major pathways for salinity-induced K^+^ loss from plant cells (Shabala *et al.*, 2016; Shabala, 2017; Adem *et al.*, 2020). Being a member of the Shaker family of transporters, the GORK channel possesses strong voltage gating and is activated upon membrane depolarization (Véry *et al.*, 2014; Shabala *et al.*, 2016). A strong correlation between genotypic ability to maintain negative membrane potential and cytosolic K^+^ retention was reported for many species (Volkov *et al.*, 2004; Chen *et al.*, 2005; Cuin *et al.*, 2008). *GORK* transcript levels are also up-regulated in salt-affected plants (Adem *et al.*, 2014; Chakraborty *et al.*, 2016). Thus, the fewer *GORK* copies a plant possesses, the more negative the membrane potential is, and the lower will be cytosolic K^+^ loss (hence, higher tolerance).

To further explore this issue, we have compared the number of orthologs between tetra- and hexaploid wheat species. The latter two (durum wheat and wild emmer) possessed ⁓3.5-fold more GORK orthologs compared with AABBDD plants (bread wheat and spelt; Fig 1). Electrophysiological studies also revealed that higher K^+^ loss from mesophyll was associated with an increased rate of (vanadate-sensitive) H^+^ pumping (Wu *et al.*, 2014). As H^+^-ATPase is considered an electrogenic process establishing cell membrane potential (Falhof *et al.*, 2016), these finding may be interpreted as an attempt by plants to restore membrane potential under conditions of soil salinity. However, GORK channels can be activated not only by membrane depolarization but also by reactive oxygen species (ROS; Demidchik *et al.*, 2010). If the latter component dominates, the activation of H^+^-ATPase may restore membrane potential but fail to prevent K^+^ loss, thus depleting a pool of available ATP via a futile cycle. It was shown that in wheat, chloroplast-generated ROS dominated NaCl-induced K^+^ efflux from leaf mesophyll (Wu *et al.*, 2015a). NADPH oxidase is another source for apoplastic ROS production. Consistent with this notion was the observation that AABB plants possess much larger (2.5-fold) number of Respiratory Burst Oxidase Homolog (RBOH) orthologs encoding NADPH oxidase as compared with plants with AABBDD genome (Fig 1).

Experiments with excised leaves also suggested that more salt-sensitive durum wheat genotypes possessed an overall higher tissue tolerance than bread wheat (Wu *et al.*, 2015b). When plants were grown in pots, leaf Na content was higher in durum compared with bread wheat (3.35±0.12 versus 2.6±0.08 mmol g^−1^ DW, respectively; Wu *et al.*, 2015b). However, no such difference in Na content was detected in experiments with excised leaves when Na^+^ was added directly to the transpiration stream bypassing Na^+^ exclusion mechanisms in roots, and the overall mesophyll cell viability was higher in salt-exposed excised durum wheat leaves. These data were interpreted as a compensation mechanism aimed at protecting the more sensitive durum wheat from the lack of effective Na^+^ exclusion in roots and/or control of xylem Na^+^ loading. Consistent with this notion, the amounts of NHX and V-PPase orthologs are much higher in more sensitive AABB tetraploid species (a 3- to 4-fold difference; Fig 1). Both are recognized as key components of the tissue tolerance mechanism, with NHXs being Na^+^/H^+^ exchangers depositing a toxic Na^+^ load in vacuoles, and V-PPase providing energy for their operation (Shabala *et al.*, 2020). Also slightly higher was the number of V-ATPase orthologs in AABB plants (Fig 1).

### A need for a closer examination of the DD genome

By screening a core collection of 179 *Aegilops* and *Triticum* accessions, Ahmadi *et al.* (2018) reported that shoot K^+^ content was ranked in the following order: *Ae. caudata* (CC)>*Ae. tauschii* (DD)>*T. boeoticum* (A^b^A^b^)>*Ae. umbellulata* (UU)>*T. urartu* (A^u^A^u^)>*Ae. speltoides* (BB). As DD plants also possessed a superior ability for Na^+^ exclusion, the leaf K^+^/Na^+^ ratio (a key determinant of salinity tolerance; Hauser and Horie, 2010) in diploid species was ranked DD>CC>UU>A^b^A^b^> BB>A^u^A^u^. This makes *Ae. tauschii* a highly attractive model species to be used in future studies. When bread wheat (*T. aestivum*) was exposed to 150 mM NaCl treatment for 3 weeks, 38% and 55% reductions in root and shoot dry weight, respectively, were observed. No significant effect, however, was observed in *Ae. tauschii* (Pour-Aboughadareh *et al.*, 2024), and more severe prolonged 200 mM NaCl treatment reduced shoot biomass in *Ae. Tauschii* by a mere 17%. In addition, in *Ae. tauschii* salt-induced K^+^ loss was less than 10% in both leaves and roots, and Na^+^ accumulation was 10 mg g^−1^ and 32 mg g^−1^ (5- and 3-fold higher than control) in root and leaf, respectively (Mansouri *et al.*, 2019). Such responses were attributed to significant up-regulation of key genes controlling plant Na^+^ and K^+^ homeostasis including *HA11*, *SOSs*, *NHXs*, *HKTs*, *KAT1*, and *HAK10* (Yang *et al.*, 2014; Mansouri *et al.*, 2019; Pour-Aboughadareh *et al.*, 2024). Salinity stress also induced *Ae. tauschii* NHX1 transcript levels for sequestration of Na^+^ in mesophyll vacuoles (Abbas *et al.*, 2021). However, compared with wild emmer, physiological studies on *Ae. tauschii* are scarce, and this gap in knowledge should be filled in the future studies.

## Conclusions and future prospects

Wheat is a major staple food in the human diet but its production under current climate scenarios is problematic, giving the extent of land salinization and the fact that wheat is highly sensitive to soil salinity. Numerous attempts have bene made to improve its salinity tolerance but progress in the field has been rather modest and insufficient when taking into account future climate scenarios. In our view, this lack of progress is a result of repeated attempts to target one specific trait—namely, Na^+^ exclusion following uptake—by either enhanced Na^+^ expulsion from the root (by SOS1) or its retrieval from the shoot (by HKT1). This strategy of excluding Na and pumping it back into the rhizosphere results in a progressive build-up of Na in the root zone, creating salinity ‘hot spots’ in the immediate proximity of the root, compromising its ability to take up water (Liu *et al.*, 2020). Overcoming this issue will require a significant investment of carbon for osmotic adjustment purposes that will come with massive yield penalties. Relying on Na^+^ as a cheap osmoticum appears to be more promising, as the carbon cost of vacuolar Na^+^ sequestration is 10-fold cheaper than production of organic osmolytes (Shabala, 2013). Thus, targeting tissue tolerance traits is much more promising, in our view. The latter will require a better understanding of molecular mechanisms involved in vacuolar Na^+^ sequestration and its retention in the vacuole, as well as cytosolic K^+^ retention. To gain such understanding, closer attention should be given to the DD genome. As discussed above, DD plants possess superior K^+^ retention traits, in addition to the ability to exclude Na^+^. The DD genome seems to be the major determinant of differential salinity stress tolerance between durum and bread wheat but, to our great surprise, in-depth studies on mechanisms of its salinity stress tolerance are rather rudimentary.

Another promising aspect is related to the structure–function relationships of numerous orthologs for some key genes regulating plant ion homeostasis and the modes of their regulation. Taking GORK channels as an example, why do tetraploid wheat species contain so many copies of GORK orthologs (Fig. 1)? Discovered first in guard cells (hence, the name—Guard Cell Outward Rectifying K^+^), this channel plays a critical role in stomatal opening and closure (Hedrich and Shabala, 2018). What is its role in roots? Does it operate as a ‘safety valve’ restoring (otherwise depolarized) membrane potential of epidermal root cells (e.g. playing a charge balancing role)? Or is it involved in salt stress sensing, as proposed elsewhere (Shabala *et al.*, 2015; Wegner *et al.*, 2025)? Do voltage and ROS sensitivity of GORK channels differ between different isoforms, and if so how does this affect root K^+^ homeostasis and stress signaling, given the emerging role of K^+^ as a new second messenger (Shabala, 2017; Rubio *et al.*, 2020)? All these questions are waiting to be answered in future studies.

## Supplementary data

The following supplementary data are available at *JXB* online.

Fig. S1. Evolution of wheat. Based on da Silva (2014).

eraf152_suppl_Supplementary_Figures_1
