# Supplementary material for: Salinity tolerance in wheat: rethinking the targets
Source: J Exp Bot. 2025 Apr 9;77(9):2666–76. doi: 10.1093/jxb/eraf152 (PMC13139655; doi:10.1093/jxb/eraf152)
Supplement: eraf152_suppl_Supplementary_Figures_1 [file eraf152_suppl_supplementary_figures_1.pptx]

## Slide 1
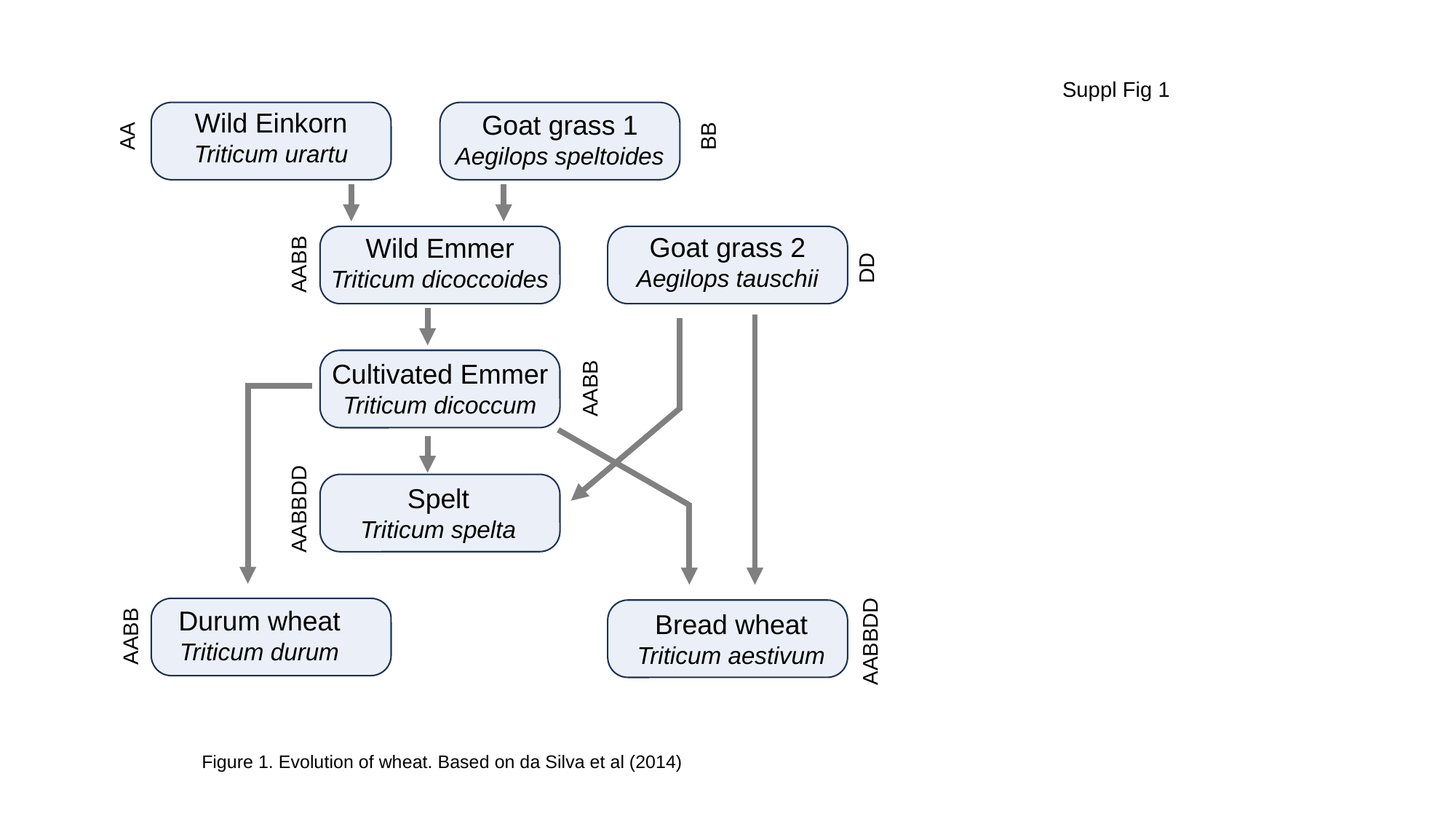

Suppl Fig 1
Wild Einkorn
Triticum urartu
Goat grass 1
Aegilops speltoides
AA
BB
Goat grass 2
Aegilops tauschii
Wild Emmer
Triticum dicoccoides
AABB
DD
Cultivated Emmer
Triticum dicoccum
AABB
Spelt
Triticum spelta
AABBDD
Durum wheat
Triticum durum
Bread wheat
Triticum aestivum
AABB
AABBDD
Figure 1. Evolution of wheat. Based on da Silva et al (2014)
